# Systemic Embolization as the Initial Presentation of a Rare Cause of Infective Endocarditis

**DOI:** 10.7759/cureus.8472

**Published:** 2020-06-06

**Authors:** Sana Riaz, Parth J Sampat, Alisha Khan, Dmitriy Bakrukov, Robert Carhart

**Affiliations:** 1 Internal Medicine, State University of New York Upstate Medical University, Syracuse, USA; 2 Radiology, State University of New York Upstate Medical University, Syracuse, USA; 3 Cardiology, State University of New York Upstate Medical University, Syracuse, USA

**Keywords:** septic brain embolism, infective endocarditis, acute limb ischemia, streptococcus dysgalactiae, transesophageal echocardiogram, aortic valve insufficiency

## Abstract

Infective endocarditis is a rare disease and is associated with a high mortality rate. The following case describes a 56-year-old gentleman who presented to our hospital with a pulseless left leg concerning for acute limb ischemia, which was managed with emergent revascularization. His subsequent workup revealed IE due to a rare organism known as *Streptococcus dysgalactiae.* Through this case, we want to showcase this rare cause of IE and also highlight systemic embolization as its possible initial presentation.

## Introduction

Infective endocarditis (IE) is a rare and life-threatening disease. There has been an increase in the incidence of IE in the previous decade, with the incidence being 15 per 100,000 population in 2011 in the United States [1]. There has also been an increase in the incidence of streptococcal endocarditis [1]. The most common organism associated with IE is recognized as *Staphylococcus aureus*, with this organism representing 31% of all IE cases [2]. Of the streptococcal species,viridans group accounts for most cases of IE. Other streptococcal species are a cause of endocarditis for less than 6% of all endocarditis cases [2].

The most common presenting feature of endocarditis is fever (96%), with the vascular embolic event being a presenting feature in less than 17% of the cases [2]. Embolization to the peripheral arteries has been observed in only 2.6% of endocarditis cases [3]. The mortality rate of IE remains high at 9.8% [4]. Peripheral arterial emboli as a result of IE can be catastrophic. Early recognition and management of IE remains crucial.

We present a case of acute IE with the initial presentation of systemic embolization from a rare cause of IE, *Streptococcus dysgalactiae.*

## Case presentation

A 56-year-old gentleman with a past medical history of untreated hepatitis C infection, osteomyelitis with right fourth toe amputation (wound cultures positive for beta-hemolytic G streptococci), and alcohol abuse (12 beers per day) presented with paresthesias and a pulseless left leg. He was noted to be febrile (102.9^⸰^F), normotensive, tachycardic (117 beats/minute), and saturating well on room air.

**Figure 1:**
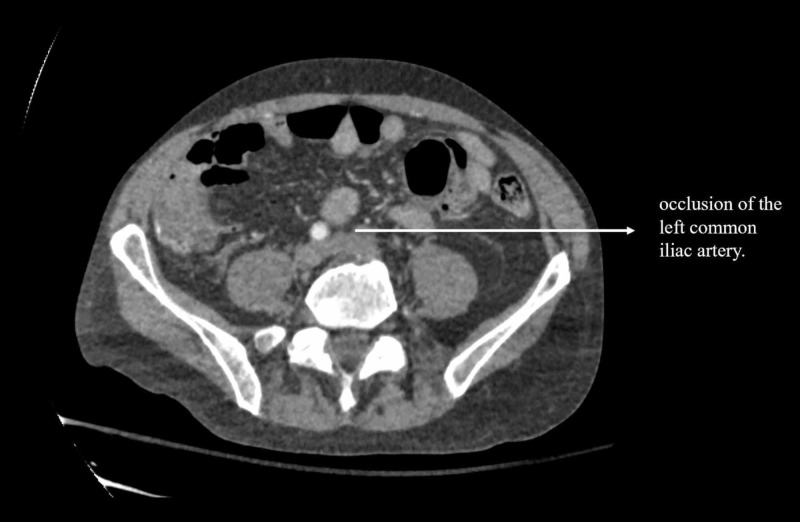
CT of the abdomen and pelvis with contrast demonstrating occlusion of the left common iliac artery.

**Figure 2:**
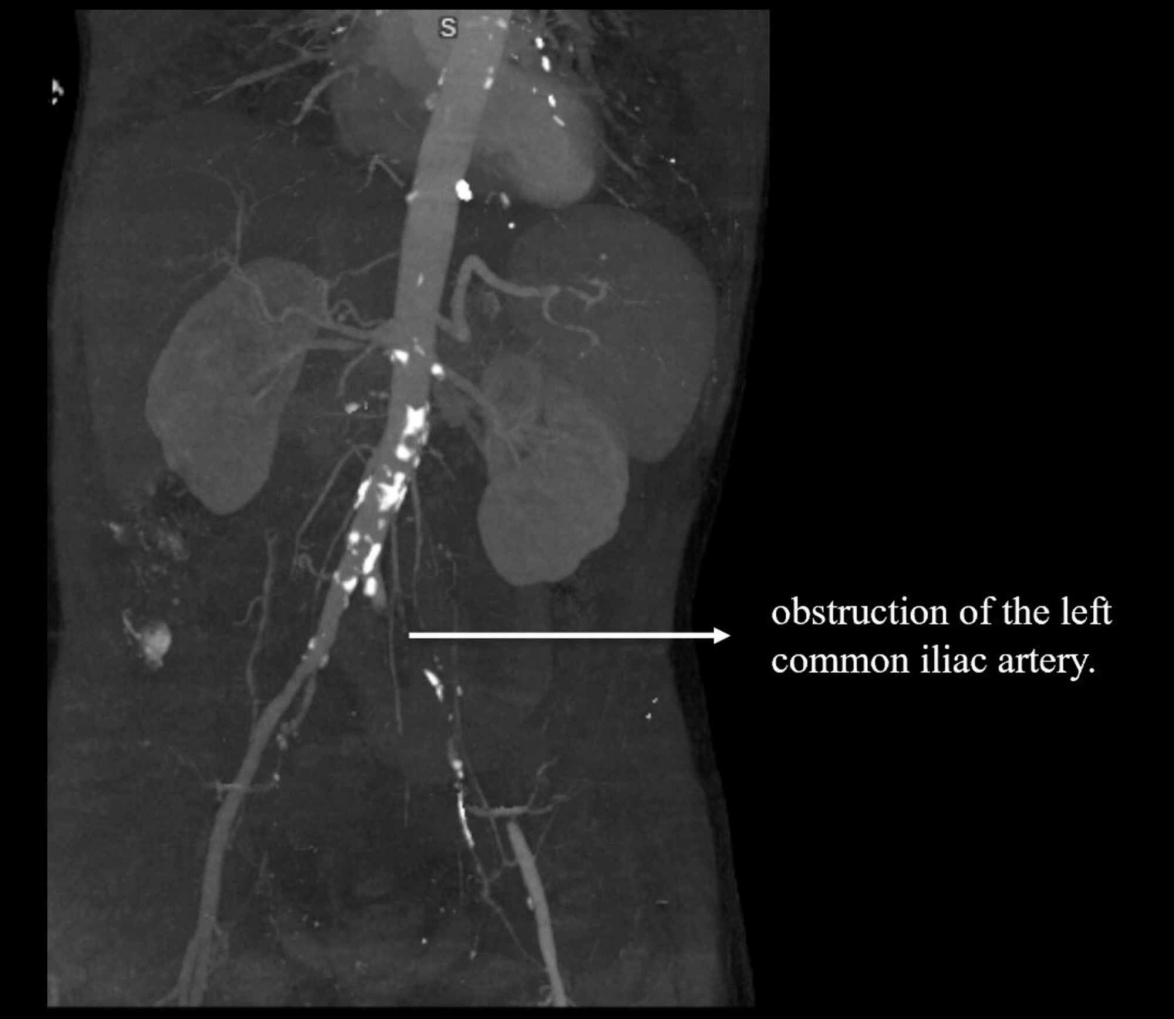
CT maximum intensity projection (MIP) demonstrating occlusion of the left common iliac artery.

Lab work was significant for normocytic anemia, leukocytosis (initially 13.5×10^3^/uL and later peaked at 33×10^3^/uL), thrombocytopenia (70×10^3^/uL), international normalized ratio <1.5, and creatinine 1.22 mg/dL. Two sets of blood cultures were positive for *Streptococcus dysgalactiae. *CT angiogram of the left limb (Figures 1, 2) showed left common iliac, external iliac, and proximal internal iliac artery occlusions. He underwent emergent revascularization with left iliac thrombectomy and was subsequently started on intravenous antibiotics, gentamicin for 10 days and ceftriaxone for six weeks.

**Figure 3:**
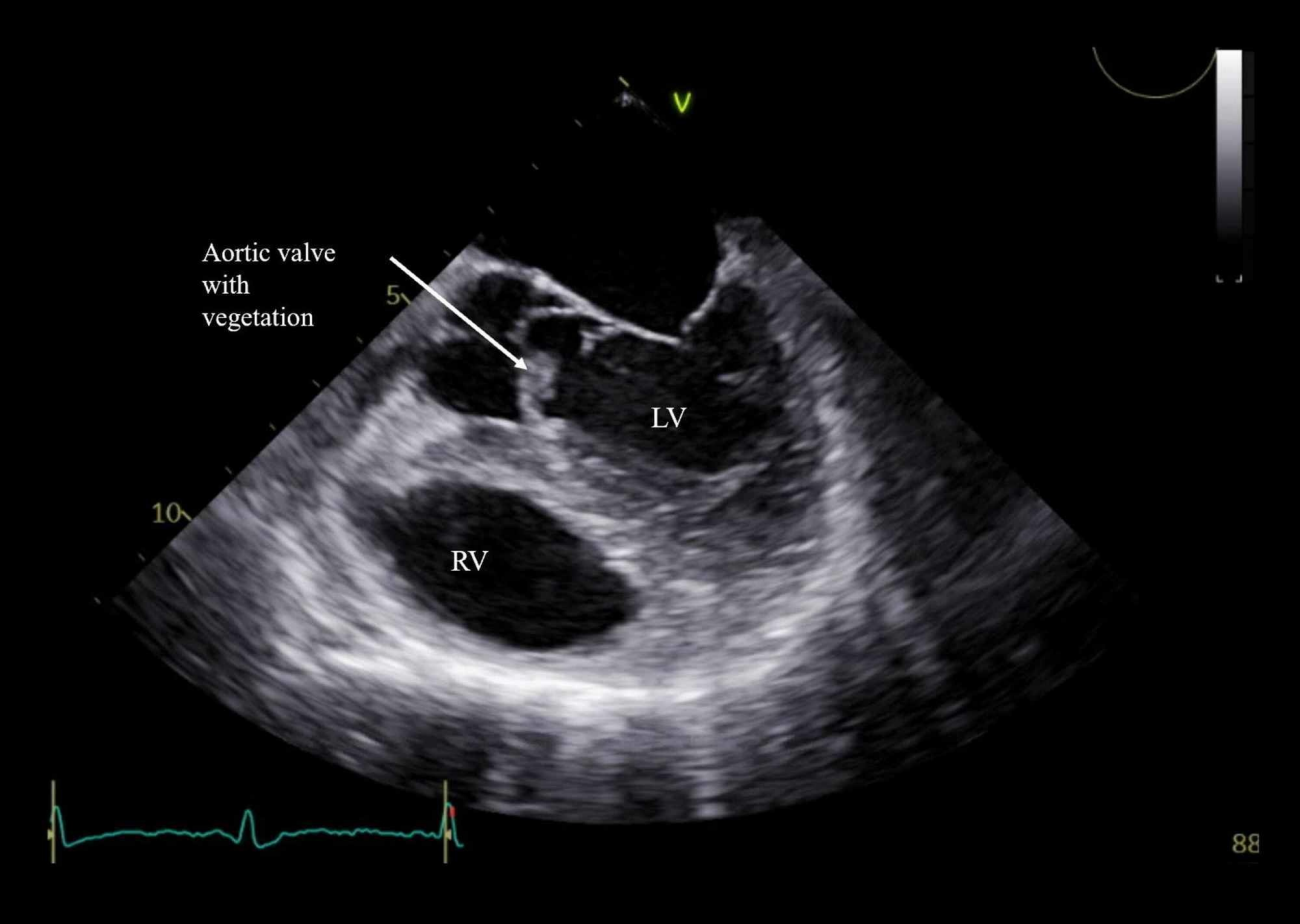
Transesophageal echocardiogram demonstrating vegetation on the aortic valve. LV: left ventricle; RV: right ventricle

Given his presentation with acute limb ischemia and septic shock, an IE workup was initiated. Transthoracic echocardiogram showed normal ejection fraction and mobile vegetation on the aortic valve along with severe aortic regurgitation (Figure 3). Findings were confirmed on a transesophageal echocardiogram. MRI of the thoracic and lumbar spine was unremarkable, but MRI of the brain showed septic emboli in bilateral cerebral hemispheres and the left cerebellar hemisphere. CT angiogram of the head and neck was negative for a mycotic aneurysm. He was evaluated by cardiothoracic surgery, and he underwent aortic valve replacement. Upon hemodynamic stabilization, he was discharged to the rehabilitation center, one month after his initial hospitalization.

## Discussion

*Streptococcus dysgalactiae* is not considered to be a typical cause of IE [5]. *Streptococcus dysgalactiae* subspecies *equisimilis *(SDSE) is a human pathogen that belongs to Lancefield groups C and G, which forms large beta-hemolytic colonies on blood agar [6]. Many clinical diseases have been associated with group C and group G streptococci, including pharyngitis, skin infections, soft tissue infections, as well as invasive infections, such as bacteremia, endocarditis, osteomyelitis, toxic shock syndrome, and others [7]. Our case demonstrates a rare cause of acute IE caused by *Streptococcus dysgalactiae.*

Systemic embolization of vegetation occurs in 22%-50% of cases with IE, with the highest embolic complications seen in people with the involvement of the mitral and aortic valves with *Staphylococcus aureus, Candida *species*, *HACEK organisms(namely *Haemophilus *species*, Aggregatibacter actinomycetemcomitans, Cardiobacterium hominis, Eikenella corrodens*, and *Kingella kingae*), and *Abiotrophia *organisms [8]. In a study of 384 patients, Thuny et al. found that only 10 cases (2.6%) presented with embolization to peripheral arteries and 62 cases (16.1%) presented with embolization to the central nervous system [3]. Our case demonstrates a patient with septic embolization to both the central nervous system and the peripheral arteries, which is a rare phenomenon occurring due to a rare organism.

An echocardiogram is an important tool in the diagnosis of IE. Transthoracic echocardiogram and, if high suspicion is present, a transesophageal echocardiogram can be obtained to demonstrate the presence of an oscillating intracardiac mass, annular abscess, prosthetic valve dehiscence, or a newly developed valvular regurgitation, which fulfill the major criteria for IE [1]. The diagnosis of IE is based on the modified Duke criteria [9]. The diagnosis of endocarditis in our patient was confirmed with one major criterion of an echocardiogram showing vegetations on the aortic valve and three minor criteria of fever >38⸰C, the vascular phenomenon of arterial and cerebrovascular embolization, and blood culture growing an organism that does not fulfill the major criteria.

Based on the guidelines of the American Heart Association, surgical intervention is essential in patients with persistent vegetation after embolization, >10 mm size of the anterior mitral leaflet vegetation, one or more embolic events during the first two weeks of antimicrobial therapy, or an increase in vegetation size despite therapy as a potential need for surgical intervention [1]. The other indications are aortic or mitral insufficiency with signs of heart failure unresponsive to medical therapy or signs of valve perforation or rupture [1]. Our patient had features of acute aortic insufficiency with acute heart failure, in addition to systemic embolization, due to which surgical intervention was warranted.

## Conclusions

Through this case, we hope to highlight the diversity in initial presentations of IE, especially when caused by an atypical organism such as *Streptococcus dysgalactiae.* We also want to emphasize the importance of prompt recognition and management of IE. Despite multiple comorbidities, the underlying infectious source was controlled by surgical intervention in our patient leading to stabilization and ultimately increasing longevity.
